# Microglia-neuron interactions in schizophrenia

**DOI:** 10.3389/fncel.2024.1345349

**Published:** 2024-03-06

**Authors:** Sophia-Marie Hartmann, Johanna Heider, Richard Wüst, Andreas J. Fallgatter, Hansjürgen Volkmer

**Affiliations:** ^1^Molecular Neurobiology, Department of Pharma and Biotech, NMI Natural and Medical Sciences Institute at the University of Tübingen, Reutlingen, Germany; ^2^Department of Psychiatry, Tübingen Center for Mental Health (TüCMH), University of Tübingen, Tübingen, Germany

**Keywords:** schizophrenia, inflammation, synaptic pruning, complement system, microglia, neuron, co-culture

## Abstract

Multiple lines of evidence implicate increased neuroinflammation mediated by glial cells to play a key role in neurodevelopmental disorders such as schizophrenia. Microglia, which are the primary innate immune cells of the brain, are crucial for the refinement of the synaptic circuitry during early brain development by synaptic pruning and the regulation of synaptic plasticity during adulthood. Schizophrenia risk factors as genetics or environmental influences may further be linked to increased activation of microglia, an increase of pro-inflammatory cytokine levels and activation of the inflammasome resulting in an overall elevated neuroinflammatory state in patients. Synaptic loss, one of the central pathological hallmarks of schizophrenia, is believed to be due to excess removal of synapses by activated microglia, primarily affecting glutamatergic neurons. Therefore, it is crucial to investigate microglia-neuron interactions, which has been done by multiple studies focusing on post-mortem brain tissues, brain imaging, animal models and patient iPSC-derived 2D culture systems. In this review, we summarize the major findings in patients and *in vivo* and *in vitro* models in the context of neuron-microglia interactions in schizophrenia and secondly discuss the potential of anti-inflammatory treatments for the alleviation of positive, negative, and cognitive symptoms.

## 1 Introduction

### 1.1 Schizophrenia spectrum disorders

Schizophrenia spectrum disorders (SCZ) are complex and heterogeneous neuropsychiatric diseases, which can manifest in a variety of symptoms, that typically emerge in late adolescence (Gogtay et al., 2011). The symptoms can be categorized into three main domains: positive symptoms (hallucinations, delusions, paranoia and thought disorder), negative symptoms (anhedonia, avolition, social withdrawal and thought poverty), and cognitive symptoms (deficits in attention, working memory and executive function (Tandon et al., 2013). Imaging studies have demonstrated structural brain changes, such as a reduction of gray matter volume in prefrontal areas, in brain tissue of SCZ patients (Mubarik and Tohid, 2016). On a cellular level, a reduction of synaptic and dendritic markers, neuronal misallocations and aberrant neurotransmitter signaling of dopamine, glutamate, and GABA have been reported (Wong and Van Tol, 2003; Osimo et al., 2019). In addition to these neuronal pathophenotypes, increasing evidence suggests a role for aberrant neuroinflammatory regulation by microglia, which are a crucial part of the cerebral immune response. An increased state of inflammation has been identified in patients with neuropsychiatric disorders with high levels of plasma pro-inflammatory cytokines, as well as chemokines found in peripheral blood and cerebrospinal fluid (Bauer and Teixeira, 2019). PET imaging studies in patients with SCZ showed elevated microglia activity patterns in regions such as the prefrontal cortex, which supports the hypothesis of increased neuroinflammation and activation of microglia in the development of neuropsychiatric diseases (Bloomfield et al., 2016).

### 1.2 Functions of microglia in the developing and adult brain

Microglia, the innate immune cells of the brain, originate from the yolk sac and populate the human brain during embryonic development (Bian et al., 2020). Under normal conditions, microglia are in a resting state and continuously survey their microenvironment to monitor the brain parenchyma (Orihuela et al., 2016). In the developing and adult brain, microglia mediate the response to local inflammation, as well as viral and bacterial infection, by release of inflammatory cytokines such as IL-6 and TNF-α (Kettenmann et al., 2011). Activated microglia become polarized towards a pro-inflammatory M1 state, undergo morphological changes, interact with complement proteins, release reactive oxygen species, and phagocytose cellular debris or pathogens in response to injury or inflammation (Hanisch and Kettenmann, 2007; Woodburn et al., 2021). Besides their involvement as part of the CNS immune response, microglia play a central role in sculpting the formation of neuronal circuits during early postnatal development. Initially, excessive numbers of synapses are formed, of which redundant and weak synaptic connections are selectively eliminated by microglial phagocytosis. This process is called synaptic pruning and is crucial to promote synaptic refinement and maturation in the developing brain (Paolicelli et al., 2011; Schafer et al., 2013). Components of the complement system, such as C1q, C4 and C3 seem to be involved in this process by marking synapses for either conservation or elimination by microglia expressing complement receptors (Schafer et al., 2012; Yilmaz et al., 2021; Cornell et al., 2022).

### 1.3 The role of microglia in schizophrenia

In SCZ, alterations in neuronal circuits seem to mainly effect the formation of synaptic connections. Recently, in an updated version of the synaptic hypothesis of SCZ, the authors postulate that synaptic loss could contribute to some symptoms of the disease (Howes and Onwordi, 2023). Data from PET imaging, iPSC, GWAS, animal and post-mortem studies suggest that microglia can be primed by genetic risk factors or early insults such as maternal infection, so that they are more susceptible to stressors later in life. In SCZ, excess activation of primed microglia might ultimately result in aberrant synaptic pruning (Howes and Onwordi, 2023). A further aspect which might play a crucial role in disease development is the shaping of an immune memory due to previous infection. In this line, it has been shown that previously activated microglia can respond more strongly to, for example, a new systemic inflammation (Schroder and Krueger, 2006; Perry, 2010) and through this may have fundamental impact on a possible development of brain diseases (Khandaker et al., 2015).

As outlined above, the study of microglia-neuron interactions could significantly contribute to our understanding of SCZ disease mechanisms and symptom development. In this review, current findings from patient, animal, and iPSC-based studies of microglia-neuron interactions in SCZ will be summarized.

## 2 Microglia-neuron interactions in schizophrenia patients

### 2.1 Post-mortem studies

Since the beginning of studies in medicine, post-mortem analysis has been a valuable tool in terms of pathological understanding, particularly in brain diseases (Rothenberg, 2008). In SCZ, investigations on post-mortem tissue have provided insights into inflammatory processes in the brain and for microglial alterations (Rodrigues-Neves et al., 2022). In this part, recent advantages in understanding the role of microglia in SCZ are reviewed, thus dividing it into a section of analysis on cellular level and a section focusing on molecular methods.

Analyzing microglia on a cellular level using ionized calcium-binding adaptor molecule-1 (Iba1) immunohistochemistry, the majority of studies focus on microglia density, mostly detecting an increase while looking at the prefrontal cortex (PFC), the temporal cortex and the hippocampus (Wierzba-Bobrowicz et al., 2005; Gober et al., 2022). In contrast to the above-mentioned findings, two publications show no differences between healthy controls (HC) and SCZ patients in the PFC (Hercher et al., 2014; Snijders et al., 2021).

Gober et al. performed a profound cellular study on a samples group of 16 SCZ patients and 13 HC, looking at frontal, temporal and cingulate cortical gray matter, subcortical white matter regions as anterior corpus callosum for microglia density, distribution, soma size and arborization patterns. Using Iba1 staining, their findings showed an increase of microglia density in all cortical gray matter regions, as well as for the frontal and temporal lobe of the subcortical white matter regions. Morphological characterizations demonstrated an increase in soma size depending on the region and a decrease in microglia arborization using a combination of Sholl analysis and 3D reconstruction (Gober et al., 2022).

In another structural analysis, Uranova et al. focused on studying satellite microglia, a subset of microglial cells, which establish a direct soma-to-soma connection with neurons and therefore are thought to be essential for neuroplasticity via the regulation of neuronal activity (Cserep et al., 2020). For this study, the PFC layer V of 21 SCZ patients and 20 HC was analyzed, checking for density and morphological changes. Interestingly, a subgroup of young SCZ patients with an illness duration of less than 26 years showed a significant increase in density compared to controls. Additionally, a lower volume fraction and reduced number of mitochondria were described in SCZ patients. In turn, a higher volume fraction and number of lipofuscin granules and vacuoles in the endoplasmatic reticulum in satellite microglia were observed in SCZ patients. These cellular changes seemed to progress with disease duration and age (Uranova et al., 2023).

Besides investigating microglial morphology, post-mortem studies offer the opportunity for a quantitative examination of factors that potentially mirror the functional status of microglia. These factors include the measurement of local concentrations of inflammatory mediators. It is worth noting that while microglia are a source of these substances, other cell types within the brain also possess the capacity to generate and release them. However, the indicators of cellular physiology are understandably compromised by the processes of cell demise and the underlying pathological changes.

In addition to cellular studies, molecular analysis of inflammatory signaling processes or pathways can back up structural findings. Aiming to prove a linkage between microglia and an increase in inflammatory signaling in SCZ, Zhu et al. examined mRNA levels of cytokines related to inflammatory processes. According to the mRNA expression of SERPINA3, IL-1β, IL-6 and IL-8, the cohort of post-mortem tissues consisting of 72 SCZ patients and 69 HC were each sub-grouped into two groups with different inflammation states, a high and low inflammation group for SCZ and a high and low inflammation group for HC. Trying to analyze the impact of microglia and macrophages on inflammatory processes, they showed unchanged levels of microglial mRNA of Iba1, Hexb and decreased CD11c mRNA in the high inflammatory subgroup of SCZ patients. At the same time, the mRNA level of macrophage marker CD163 was increased in the high inflammatory group of SCZ, even compared to the low inflammation subgroup of SCZ patients. Secondly, pro-inflammatory macrophage marker CD64 was also elevated and more related to CD163 mRNA in the high inflammatory subgroup of patients, while macrophage recruitment chemokine CCL2 mRNA was increased in SCZ and positively linked to CD163 mRNA, thus highlighting an increase of pro-inflammatory macrophages in SCZ (Zhu et al., 2022).

Examinations on post-mortem tissues have contributed in the case of SCZ to many groundbreaking concepts and an improved pathological understanding.

### 2.2 Brain imaging studies

Over recent years, a notable progress in neuroimaging techniques has been achieved, thus leading to new *in vivo* insights in the field of brain research. Besides other imaging techniques, which so far lack the possibility of a direct detection of inflammatory processes, we will focus here on studies using positron emission tomography (PET). In terms of PET and inflammation the existing methodologies primarily focus on a mitochondrial protein, specifically the 18-kDa translocator protein (TSPO), which is prominently expressed in activated microglia, possibly triggered by pathological circumstances.

For better understanding it makes sense to divide the available data up, for example by the used tracers, the way of calculating TPSO binding, or as done in our case, in different disease stages, namely into first episode or recent onset and a longer period or chronical state.

Data on recent onset forms or first psychotic episodes seems contradictory, describing increases and decreases or the absence of changes in TSPO tracing (Collste et al., 2017; Hafizi et al., 2018; Ottoy et al., 2018). Ottoy et al. described an increase in TSPO binding in several brain regions, Collste et al. found a significant decrease in partly the same brain regions, while Hafizi et al. described no significant changes in a cohort of untreated patients. All mentioned studies used different tracers.

Looking at a chronic state of disease, one has to keep in mind that patients are mostly under anti-psychotic treatment. This together with other confounding factors may reflect the conflicting data. Above-mentioned authors describe an increase, while for example Di Biase and other authors found normal levels of TSPO binding (Kenk et al., 2015; Di Biase et al., 2017).

Other studies match their findings from imaging with additional data. In a cohort of 12 SCZ patients with a recent onset, compared to 14 HC, Coughlin et al. quantified TSPO levels in several brain regions and subsequently correlated their findings with IL-6 levels from plasma and CSF. While no changes of TSPO binding were described, the patients with recent onset of SCZ showed a significant increase in IL-6, both in plasma and CSF, thus suggesting that increased levels of IL-6 may occur in the absence of changed TSPO signal and this might be linked to medication (Coughlin et al., 2016).

Using a second generation tracer on a group of patients with a high-risk of psychosis and a cohort with a longer existing history of disease, Bloomfield et al. report an increase in TSPO signal for both groups, but in contrast to other studies, they were able to link their findings to symptom severity on imaging day using PANSS (Positive and Negative Syndrome Scale) and BDI (Beck Depression Inventory) (Bloomfield et al., 2016). The main findings of post-mortem and imaging studies are summarized in Table 1.

**TABLE 1:** Brain imaging studies of microglial activation in SCZ patients.

Model	Method	Region/Material	Samples	Focus	Description (SZC vs HC)	References
Post-mortem	– IHC	– frontal + temporal lobe	9 SCZ + 6 HC	– MHCII staining	–↑ number of activated microglia –↑ microglia damage	Wierzba-Bobrowicz et al., 2005
Post-mortem	– IHC – 3D reconstruction	Grey matter – frontal, temporal, cingulate – subcortical white matter / SCWM	16 SCZ + 13 HC	– Iba1 staining	– microglial density ↑ grey matter – microglial density ↓ /SCWM – microglia soma size ↑ – microglia arborization ↓ – association: age + ↓ arborization	Gober et al., 2022
Post-mortem	– IHC	– gray matter	37 SCZ + 40 HC	Microglia marker	–↑ Iba1, ↑ CD64/HLA-DR	De Picker et al., 2021
Post-mortem	– IHC – Protein quantification		20 SCZ + 20 HC	– GFAP / Iba1 staining – CNPase immuno blot	– microglia density unchanged – astrocyte density unchanged	Hercher et al., 2014
Post-mortem	– IHC – Quantitative PCR	– temporal cortex	12 SCZ + 16 HC 9 SCZ + 14 HC	– Iba1 staining – Microglia Gene expression	–↓ microglia genes (temporal+frontal) – microglia genes unchanged (sub-cortex)	Snijders et al., 2021
Post-mortem	Electrone microscope	Layer5 PFC	21 SCZ + 20 HC	Satelite Microglia (SatMg)	– SatMg density ↑ – Mito volume fraction (vf) ↓ – Mito number (n) ↓ – lipofusin granules vf ↑ – lipofusin granules n ↑ – vacuoles in ER ↑	Uranova et al., 2023
Post-mortem	Quantitative PCR	PFC	72 SCZ + 69 HC – subgrouped high inflam + low inflam SCZ high inflam + low inflam HC	Microglia mRNA level	– Iba1-, Hexb levels unchanged – CD11c ↓, CD106, IL-10 unchanged / ↓ – CD 163 ↑, CCL2 ↑	Zhu et al., 2022
Imaging	TSPO - PET Scan	PFC, ACC, PC	16 SCZ + 16 HC	[^11C^]-(R)-PK11195	– TSPO binding unchanged: unmedicated vs HC –↑ TSPO binding – TSPO correlation with PANSS	Holmes et al., 2016
Imaging	TSPO - PET Scan	– gray matter – genotype	11 SCZ + 17 HC	[^18^F]-PBR111	–↑ neuroinflammation – TSPO binding age-dependent	Ottoy et al., 2018
Imaging	TSPO - PET Scan	– Hippocampus –gray matter	7 SCZ + 8 HC	[^11C^]-(R)-PK11195	– Hippocampus: neuroinflammation ↑ (binding potential) – grey matter: unchanged binding potential	Doorduin et al., 2009
Imaging	TSPO - PET Scan	– gray matter, genotype	16 SCZ + 16 HC	[^11^C]-PBR28	– ↓ grey matter volume – clinical + cogn. unchanged	Collste et al., 2017
Imaging	TSPO - PET Scan	– Whole brain, grey matter	19 SCZ + 20 HC	[^18^F]-FEPPA	– volume	Hafizi et al., 2017
Imaging	– TSPO - PET Scan – Immunoasay	gray matter	15 SCZ + 27 HC	[^11C^]-(R)-PK11195	– TSPO binding unchanged – IL6 unchanged	Di Biase et al., 2017
Imaging	TSPO - PET Scan	– Hippocampus – mPFC, DLPFC – striatum, cc, cingulum – genotype	18 SCZ + 27 HC	[^18^F]-FEPPA	– microglia activation unchanged (gray + white)	Kenk et al., 2015
Imaging	TSPO - PET Scan IL6 plasma + CSF	– cortical – subcortical	14 SCZ + 16 HC	[^11^C]DPA-713	– microglia activation unchanged – correl. IL6 plasma + CSF	Coughlin et al., 2016
Imaging	TSPO - PET Scan	– gray matter – genotype	14 SCZ + 14 HC	[^11^C]-PBR28	–↑ neuroinflammation – no correlation TSPO + PANSS	Bloomfield et al., 2016

### 2.3 Body fluids/genetics

Further evidence of a link between inflammation and SCZ arise from studies performed in body liquids, as blood or CSF. The usage and analysis of these sources bears several advantages compared to the previously mentioned systems. Due to the comparably easy access, the number of sample sizes in studies is far bigger, which in the case of SCZ is crucial for genetic analysis.

#### 2.3.1 Expression of microglia-related genes

In SCZ, a disease with a high heritability, genetical analysis seems evident. In 2011 and 2014, two big consortia published the first GWAS data for SCZ with samples sizes of over 20,000, describing among others the strongest risk factor for a variant to be found in the MHC of chromosome 6, a region important for genes of the immune system (Schizophrenia Psychiatric Genome-Wide Association Study (GWAS) Consortium, 2011). A further study showed a risk association for a region lying within complement component 4 (C4) genes, which are crucial for the immune system, too (Sekar et al., 2016). Although the C4 system is responsible for phagocytic removal of damaged cell components, and furthermore was shown to be involved in synaptic pruning (Schafer et al., 2012), no direct link between the C4 system and synaptic engulfment in humans could be established yet.

Further genetic studies were performed, among one describing a rare genetic variant in the gene of the chemokine receptor 1 (CX3CR1) (Ishizuka et al., 2017). A gene only expressed by microglia in the brain. In contrast, Trubetskoy et al. performed another GWAS study with over 70.000 samples with no coverage of the previous existing findings (Trubetskoy et al., 2022).

#### 2.3.2 Peripheral markers of inflammation

Elevated peripheral markers of inflammation found in blood or CSF have been previously associated with SCZ (Miller et al., 2011; Di Nicola et al., 2013). One of the underlying concepts behind elevated markers is a microglia-induced activation of the immune system (Bishop et al., 2022). Findings in part are still contradictory (Lizano et al., 2021, 2023), but several studies describe a connection between increased inflammatory markers, as IL-6 or CRP with symptom severity in psychosis with secondly and advanced brain aging, and further a worse response to antipsychotic treatment, impaired working memory and altered global cognitive performance and processing speed (Mondelli et al., 2015; Kose et al., 2021; Klaus et al., 2022; Morrens et al., 2022). Given not all SCZ patients show elevated inflammatory markers, the debate of inflammatory subgroups seems obvious. Using different methods such as machine learning (Enrico et al., 2023) or principal component analysis, it was thought that about 30–50% of all patients suffering from psychosis can be in included in a subgroup with elevated inflammatory markers (Tamminga et al., 2021; Bishop et al., 2022). These patients have shown to have a more impaired cognitive function than a comparable group with psychosis and a low inflammation status (Sæther et al., 2023).

Seeing that inflammation bears a strong link to SCZ, it raises the question of effects of anti-inflammatory treatments. Jeppesen et al. performed a meta-analysis looking at the effects of an add-on treatment of anti-inflammatory drugs to an antipsychotic treatment. Dividing the substances into groups of primarily anti-inflammatory (immunmodulation and monoclonal antibodies) and secondly potential anti-inflammatory properties (Neurosteroids, N-Acetylcystein, …), they were able to show a slight improvement in the PANSS Score in the treatment groups with antipsychotic (AP) plus anti-inflammatory treatment compared to AP plus placebo. Trials performed on only SCZ, not including other psychotic diseases, showed an even bigger effect, but at the same time there was no difference between the groups of anti-inflammatory drugs (Jeppesen et al., 2020). Another well conducted patient study focused on the benefit of the tetracycline antibiotic minocycline on negative symptoms by recruiting 207 patients developing SCZ within the past 5 years. Treatment of the SCZ patients with minocycline over a period of 12 months did not reveal any improvement of negative or other symptoms, nor primary biomarker outcomes such as medial prefrontal grey matter volume, dorsolateral prefrontal cortex activation during a working memory task, and plasma concentration of IL-6, differed compared to the placebo group (Deakin et al., 2018).

Although actual results from post-mortem, imaging and body fluids to date lack a clear identification of direct disease causes, there is growing evidence of a participation of the inflammatory system in the development of SCZ. The direct relation between findings and patients and the possibility of a potential biomarkers or a support in terms of decisions towards treatment or diagnosis is a big advantage. Looking at peripheral markers, mostly an altered level of inflammatory proteins is described in SCZ (Halstead et al., 2023), but still cannot be put in direct association with processes reflecting a possible inflammation in the CNS.

## 3 Microglia-neuron interactions in animal models of schizophrenia

As outlined above, post-mortem, brain imaging, genetic and biomarker studies have provided evidence for increased inflammation and microglial activation in SCZ patients. To further study the role of inflammation, the mechanisms involved, and how they might contribute to the reported neuronal phenotypes in SCZ (see 1.1), animal models of SCZ have been created. Existing rodent models aim to mimic increased inflammation and/or microglial activation. This is commonly achieved by, for example, prenatal immune challenges in maternal immune activation (MIA) models (described in 3.1) or by genetic manipulation of immune-associated risk genes such as complement component *C4* (described in 3.2).

### 3.1 Maternal immune activation (MIA) models

A commonly used model for neurodevelopmental disorders as SCZ or autism spectrum disorder is the model of maternal immune activation (MIA). MIA models were developed based on the neurodevelopmental hypothesis of SCZ to study premorbid neurocognitive alterations. According to this disease hypothesis, pre- or perinatal hazards such as brain lesions (Weinberger, 1987), prenatal exposure to viral infections (Brown, 2006) or hypoxia at birth (Lewis and Murray, 1987) can increase the risk of developing SCZ.

To induce MIA, pregnant rodents are injected with immunostimulants such as Polyinosinic:polycytidylic acid (Poly I:C) to provoke an immune response which interferes with fetal brain development. Poly I:C has been shown to induce the release of IL-10 in pregnant rodents, followed by elevation of fetal IL-1β levels (Meyer et al., 2005). Persisting changes in cytokine levels in MIA offspring have also been reported (Juckel et al., 2011). Interestingly, MIA offspring show deficits in social interaction and sensory information processing, as for example reduced pre-pulse inhibition (PPI), which can be related to SCZ symptoms (Van den Eynde et al., 2014; Giovanoli et al., 2016; Ozaki et al., 2020). These behavioral phenotypes were shown to be rescued by treatment with the tetracycline antibiotic minocycline (Xia et al., 2020).

Most studies performed with MIA models so far focus on the separate description of either microglial or neuronal phenotypes. Overall, an increase of microglial activation in response to maternal immune challenge has been reported, as indicated by increased density of Iba1+ microglia, microglia tip motility (Juckel et al., 2011; Ozaki et al., 2020) and CD68/CD11b immunoreactivity in different brain regions (Van den Eynde et al., 2014; Ozaki et al., 2020). Neuronal phenotypes which have been described in MIA offspring include altered pre- and postsynaptic numbers (Giovanoli et al., 2016), as well as reduced numbers of parvalbumin (PV)-positive neurons in the hippocampus, resulting in decreased GABAergic transmission (Xia et al., 2020). While none of these studies investigates the direct interaction of microglia and neurons, anti-inflammatory treatment in a MIA model was shown to rescue microglial activation and eventually the observed neuronal phenotypes (Xia et al., 2020). In MIA offspring treated with minocycline, microglial activation was reduced in the hippocampus and the observed reduction of PV+ neurons, impaired GABAergic transmission and γ-oscillations were rescued. These findings suggest a link between microglial activation induced by inflammation and the emergence of neuronal pathophenotypes, resulting in behavioral abnormalities. However, it remains to be investigated how exactly the two cell types interact in this context.

### 3.2 Genetic animal models

As described above, copy number variations in complement component *C4* were shown to be associated with SCZ in human patient samples (Sekar et al., 2016). Additionally, a reduction of synapse density in the brain of patients with SCZ was demonstrated in a recent PET imaging study (Onwordi et al., 2020). These and similar findings from patients have led to the formulation of the synaptic over-pruning hypothesis, which suggests excessive pruning of synapses by activated microglia in a complement-dependent manner (Germann et al., 2021). While no direct evidence supporting this theory has yet been obtained directly from patients, *C4* mutant rodent models have helped to shed some light on the underlying mechanisms.

Originally, complement-dependent synaptic pruning was described and studied in the mouse retinogeniculate system. The establishment of topographic projections from retinal ganglion cells (RGC) to the dorsolateral geniculate nucleus (dLGN) during development, is a process believed to be mediated by synaptic pruning (Stevens et al., 2007). Complement components C1q and C3 were previously shown to be involved in pruning of RGC inputs. Mouse knockout models of C1q and C3 showed deficits in eye-specific segregation in the dLGN, comparable to the effect of blocking electrical activity in these neurons (Stevens et al., 2007). Even though C1 and C3 have been linked to SCZ by some groups, the evidence so far is relatively inconsistent (Mayilyan et al., 2008). Therefore, we will focus on the role of complement component C4 in this review.

To study the involvement of C4 in synaptic pruning, Yilmaz et al. created mouse models overexpressing variants of the gene (Yilmaz et al., 2021). Mice showed altered social behavior, increased anxiety, and deficits in spatial working memory, resembling the negative symptoms of SCZ (Yilmaz et al., 2021). C4 is expressed by neurons, localized to axons, dendrites and synapses, and exists as two functionally distinct genes, *C4A* and *C4B* (Sekar et al., 2016). Human C4 variants C4A and C4B were introduced into the mouse genome and binding levels of C4A and C4B to isolated synaptosomes were studied. C4A showed higher binding levels, which was also confirmed by colocalization analysis of C4A/B and vesicular glutamate transporter 2 (vGlut2) at RGC synaptic terminals. The unique role of C4A in synaptic pruning was further confirmed by comparison of high vs. low *C4A* copy number mice. Microglia in high *C4A* mice engulfed more synaptic inputs than low *C4A* during development (P10) and showed high expression levels of activation marker CD68 at P10 and P40, suggesting that these are the critical time frames of synaptic pruning in the mouse retinogeniculate system. A reduction of synapse numbers was still observed in P180 high copy number mice, implicating that aberrant pruning during development can have long-lasting consequences in the adult brain. Finally, the behavior of C4A overexpression mice aged 10–12 weeks was also impacted by altered synapse numbers.

Overall, these studies suggest that a potential mechanism contributing to synapse loss in SCZ is the excessive pruning of synapses by microglia during a critical developmental period. Elevated inflammation in SCZ can activate microglia to prune more synaptic connections than necessary, leading to persistent synaptic deficits, potentially related to the emergence of SCZ symptoms later in life. Synaptic pruning seems to be partially mediated by components of the complement system, such as C4A, C3 and C1q, which are known risk genes for SCZ (Zakharyan et al., 2011; Sekar et al., 2016; Chen et al., 2021) and mark synapses for degradation. The study of neuron-microglia interactions is therefore crucial to uncover molecular mechanisms governing these interactions and new drug targets for the disease.

Overall, *in vivo* animal studies are useful for investigating the mechanisms of neuroinflammation in a systemic environment which can be easily accessed and manipulated. Rodent models allow site and time-specific interventions such as overexpression/knockout of SCZ risk genes or prenatal immune challenges in MIA models. These models have helped to support the notion of an increased susceptibility of patient microglia to become activated, potentially due to prenatal “priming” by maternal infections or other adverse influences during gestation. Over-activation of microglia and increased expression of complement components (e.g., C4A) could then lead to aberrant synaptic pruning in SCZ. The major findings of the described *in vivo* animal studies are summarized in Table 2.

**TABLE 2:** Studies of microglial activation and related neuronal phenotypes in SCZ animal models.

Type of model	Intervention/ treatment	Observation period	Neuronal phenotypes	Microglial (MG) phenotypes	Behavioral phenotypes	Microglia-neuron interaction	References
MIA (mouse)	PolyI:C injections at E9 (20 mg/kg)	P30	Not reported	↑ Iba1+ MG (hippocampus, striatum), ↓ MG processes and branches	Not reported	Not reported	Juckel et al., 2011
MIA (mouse)	PolyI:C injections at E12/15 (10 mg/kg)	E18/P10	Not reported	↑IL-6 expression in E18 MG, ↑ tip velocity in E18 MG, ↓ tip velocity in postnatal MG	↑ social anxiety	Not reported	Ozaki et al., 2020
MIA (rat)	PolyI:C injections at E15 (4 mg/kg)	P56/90/180	Not reported	↑OX-42 immunoreactivity, MG density, ↓arborization (corpus callosum, hippocampus, thalamus most affected)	↓ basal acoustic startle responce, ↓ spontaneous locomotor activity	Not reported	Van den Eynde et al., 2014
MIA (mouse)	PolyI:C injections at gestatational day 9 (5 mg/kg)	P35/84 (PPI), P40/90 (IHC)	↓ presynaptic SYN1 and bassoon density (hippocampus), ↓ postsynaptic PSD95, SynGap	– Iba1 + MG, branching, soma area, CD68+ MG unaltered	Adult-onset PPI deficits	Not reported	Giovanoli et al., 2016
MIA (mouse)	PolyI:C injections at gestational day 12 (5mg/kg) + Minocycline injections from P21-56 (40 mg/kg)	P56	↓ PV protein levels (hippocampus*, mPFC), ↓ PV+ cells (DG, CA1), ↓ mIPSC frequency*, ↑γ-oscillations*	↑ IL-1β, TNFα, IL-6 (hippocampus)*, ↑ Iba+ MG (hippocampus), ↑ MG ramification*	↑ hyperactivity*, ↓ PPI*, ↓ social interaction, ↓ spatial memory*	Minocycline treatment → anti-inflammatory effects on MG → allevation of neuronal phenotypes	Xia et al., 2020 *= improved by minocycline treatment
Genetic (mouse)	Overexpression of human C4A/C4B	P10, P40, P180	↓ synapse numbers in high *C4A* copy number mice (retinogeniculate system)	↑ engulfment of synaptic inputs (P10), ↑ expression of activation marker CD68 (P10, P40) in high *C4A* copy number mice	↑ anxiety, ↓ social behavior, spatial working memory	Activated microglia in high *C4A* mice → excessive pruning of synaptic terminals	Yilmaz et al., 2021

## 4 Microglia-neuron interactions in iPSC models of schizophrenia

Post-mortem studies, as well as rodent studies described in the sections above, provided little information about mutual microglia-neuron interaction. Hence, iPSC-derived model systems have been developed allowing the examination of microglia-neuron interactions in the context of neuroinflammation more precisely and help to better understand how the two cell types might contribute to the reported neuronal phenotypes observed in SCZ patients. iPSC are a promising model system to study neurodevelopmental disorders and represent a suitable tool for future regenerative and personalized medicine. One of the major hallmarks of iPSC is the capability to self-renew indefinitely and to undergo differentiation, a property called potency (Robinton and Daley, 2012). The greatest advantage of iPSC is that they originate from a specific donor and harbor the genetic background of this donor. Therefore, it is possible to differentiate patient-derived iPSC into various disease-relevant cell types aiming to remodel the respective disease *in vitro*.

### 4.1 Advantages of co-culturing microglia and neurons

So far, numerous iPSC-derived model systems have been established and provide new insight into human brain development and aberrations in early neurodevelopment leading to psychiatric diseases. One major advantage of iPSC-derived model systems is the possibility to easily co-culture desired cell types that allows the examination of cell-cell interactions more detailed. To better understand the contribution of microglia-neuron interactions in the context of SCZ, it is essential to first examine how microglia and neurons behave and interact with each other in a healthy co-culture model.

In a study from 2017, Haenseler et al. recapitulated the *in vivo* development of microglia *in vitro* using an iPSC-based model system. To this end, they generated iPSC-derived macrophage precursor cells and co-cultured them with iPSC-derived cortical neurons, to mimic the infiltration of macrophages in the developing brain. Co-culturing induced microglia with iPSC-derived neurons leads to an improved maturation state, optimized survival and functionality of both cell types (Haenseler et al., 2017). Subsequent RNA sequencing of isolated co-cultured microglia revealed the expression of the six key microglia-specific genes identified by Butovsky: MERTK, GPR34, PRS1, C1QA, GAS6, P2RY12 (Butovsky et al., 2014) and showed that co-cultured microglia had comparable expression levels of these genes with cultured adult human microglia. Furthermore, comparing co-cultured microglia and microglia in monocultures in gene ontology analysis revealed downregulation of signaling pathways such as type I interferon and toll-like receptor TLR1/TLR2 signaling pathways associated with antiviral responses and bacterial recognition (Haenseler et al., 2017). In contrast, genes upregulated in co-cultured microglia were enriched in biological processes important for microglia functions under normal conditions. To see whether co-cultured microglia are able to respond to inflammatory responses, LPS stimulation was applied, leading to cluster formation and less ramifications indicating a transition into activated microglia. Noteworthy, microglia-like cells cultured in monocultures displayed an overall higher secretion of pro-inflammatory cytokines, whereas co-cultured neurons promoted a more anti-inflammatory state indicated by the increased levels of the anti-inflammatory cytokine IL-10, found in the co-cultures (Haenseler et al., 2017).

This study underlines the relevance of using co-culture models by showing that neuron and microglia from HC influence each other in a positive way which is seen in an increased maturation state of both, as well as in dampened secretion of chemokines. Culturing iPSC-derived microglia solely leads to different properties. Overall, co-culturing microglia and neurons is a helpful approach to examine direct and mutual interactions between different cell types, especially in the face of the disease which will be discussed in the following sections.

### 4.2 Co-culturing schizophrenia neurons and microglia

In SCZ, synaptic loss and excessive synaptic pruning was already postulated reflecting abnormalities in both microglia and neurons. Co-culturing iPSC-derived neurons and microglia enables the direct investigation of mutual interactions and help to better understand the underlying mechanisms leading to increased inflammation and synapse loss observed in SCZ patients.

#### 4.2.1 Co-culturing excitatory neurons with microglia

In a recent study from Breitmeyer et al., a co-culture model of induced microglia and induced NGN2 neurons, both cell types for the first time originating from iPSC, was used to study interactions of neurons and microglia in either matching (CTR neurons + CTR microglia, SCZ neurons + SCZ microglia) or mixed pairs (CTR microglia with SCZ neurons and vice versa). Co-culturing excitatory NGN2 neurons from HC with SCZ-derived microglia resulted in a significantly decreased presynaptic density, as did co-culturing SCZ neurons with HC microglia which revealed a similar level of decrease. Furthermore, the uptake of synaptic structures by microglia cells was significantly increased in SCZ conditions which was identified by the presynaptic marker Synapsin 1 co-localizing in microglia. Again, co-cultures of SCZ neurons and microglia revealed the strongest uptake, followed by the co-culture comprising SCZ neurons and CTR microglia (Breitmeyer et al., 2023). In this study, not only co-cultures, but also monocultures of neurons and microglia were examined to identify phenotypes in SCZ cells. As already observed in the co-culture, SCZ NGN2 neurons in monoculture revealed a significant reduction of presynaptic terminals compared to CTR. Interestingly, RNA sequencing data of the iPSC-derived microglia cells derived from SCZ patients revealed significant elevation of immune-related transcripts. Upregulation of NLRP2, NLRP3 and TLR4, linked to NFκB signaling, identified the involvement of the inflammasome pathway. Further validation of an activated inflammasome in SCZ microglia was depicted in increased TNFα levels in the supernatant, increased NFκB translocation in the nucleus as well as increased caspase-1 activation (Breitmeyer et al., 2023). Altogether, this study demonstrated for the first time a co-culture model of iPSC-derived microglia and neurons and showed not only the contribution of microglial, but also the contribution of neuronal features to excessive elimination of synaptic terminals. Secondly, the study identified increased activation of the inflammasome implicating an increased inflammation state in microglia cells derived from SCZ patients.

Similar results were observed in another study that made use of a technically different co-culture model system by co-culturing neuronal synaptosomes with microglia. For this purpose, synaptic structures were isolated from iPSC-derived NGN2 neurons as reported previously (Sellgren et al., 2017) and microglia cells obtained from blood of HC and SCZ. Increased engulfment of synaptic structures with a robust uptake of the post-synaptic marker protein PSD95 as well as the presynaptic marker SNAP-25 was observed in microglia from SCZ patients. Again, matching (CTR microglia + CTR synaptosomes, SCZ microglia + SCZ synaptosomes) or mixed pairs (CTR microglia with SCZ synaptosomes and vice versa) in the co-culture revealed lowest uptake of synaptic structures in HC conditions. In further experiments, Sellgren et al., focused on whether the involvement of the complement system leads to increased engulfment of synaptic structures by microglia in the SCZ condition. Indeed, *C4A* risk variants were strongly associated with increased complement deposition on synaptic structures, whereas no significant effect was observed for *C4B* variants implementing that variability at the C4 locus may have influence on synaptic pruning. Quantification of co-localizing C4^+^ and PSD95^+^ inclusions in induced microglia confirmed the hypothesis of increased *C4A* expression and its involvement in synaptic pruning in SCZ (Sellgren et al., 2019). Additionally, in line with these findings, Breitmeyer et al. also identified the significant upregulation of the *C4A* gene in SCZ microglia.

An iPSC-derived co-culture model system of neurons and microglia can replicate the phenotypes observed in SCZ patients and help to identify underlying mechanisms. As seen in patients, the two described co-culture models also observed excessive synaptic loss and identified the uptake of synaptic structures by microglia. Both studies pinpointed that not only microglial but also neuronal features contribute to the excessive elimination of synaptic terminals. Underlying mechanisms involved in the disease phenotype such as increased inflammation and increased activation of the inflammasome seem to play a crucial role in the disease pathology. As described above, previous post-mortem and genome-wide association studies of SCZ patients showed strong association with gene variants that affect the *C4* gene showing an increased expression of *C4A*. However, no direct association between the complement system and excessive synaptic pruning in humans was developed yet. Interestingly, the iPSC co-culture studies detected the involvement of the complement system in the diseased phenotype and confirmed the link between C4A overexpression and increased synaptic loss.

#### 4.2.2 Co-culturing interneurons with microglia

Another affected neuronal cell type in SCZ, crucial for excitation-inhibition (E-I) balance in the prefrontal cortex, are cortical interneurons. In SCZ patients, cortical interneurons depict abnormalities in gamma oscillations, potentially leading to cognitive impairments (Volk and Lewis, 2014). Co-culturing of HC iPSC-derived cortical interneurons with murine immortalized BV2 microglia that were activated previously with LPS and subsequent RNA sequencing of co-cultured neurons revealed deficits in metabolic pathways and upregulation of inflammatory genes such as NFKBIZ, TNFRSF12A and TNFAIP3 compared to neurons that were co-cultured with non-stimulated microglia (Park et al., 2020). In contrast, pathway analysis of differently expressed genes did not show aberrations in the expected inflammatory response, but rather showed the involvement of the metabolic pathway as a major target confirmed by the upregulation of the metabolic regulators *CTGF* and *THBS1*. Cortical interneurons co-cultured with stimulated microglia or in contact with activated-microglia-conditioned medium led to a significant downregulation of basal respiration, maximum respiration, and ATP production. Since synapse formation and arborization of neurons is linked to vast energy consumption, direct effects of altered metabolism can lead to significant deficits (Ni et al., 2020). Comparisons between HC and SCZ cortical interneurons revealed mitochondrial dysfunction in both HC and SCZ neurons, cultured in activated- microglia-conditioned medium. Interestingly, after the removal of inflammatory stressors, HC cortical interneurons recovered, whereas SCZ neurons still exhibited disruptions in metabolic pathways indicating a persistent response.

This study suggests that there is an interaction between assumed SCZ genomic background and environmental risk factors showing the importance of prenatal immune activation which could play a key role in disturbing normal neurodevelopment even in the absence of pathogens. Unlike the two described studies before (Sellgren et al., 2019; Breitmeyer et al., 2023), this study only focuses on the neuronal phenotypes when exposed to pro-inflammatory factors rather than showing interactions between co-cultured neurons and microglia. The major findings of iPSC-derived co-culture studies comprising microglia and neurons are summarized in Table 3.

**TABLE 3:** Studies of neuron-microglia interaction in iPSC-based SCZ models.

Type of Model	Cell Sources	Assays to Study Microglia-Neuron Interaction	SCZ Microglial Phenoytpe	SCZ Neuronal Phenotype	Microglia-Neuron Interaction in SCZ	Treatment	References
**Co-culture microglia + neurons:** – HC microglia + HC neurons	– iPSC-derived microglia – iPSC-derived neurons	– RNA sequencing – gene ontology analysis	– Improved maturation state ↓ type-I interferon responses ↑ biological processes	– Improved maturation state	– Anti-inflammatory state ↑ cytokine IL-10	No treatment	Haenseler et al., 2017
**Co-culture microglia + NGN2 neurons:** – HC microglia + HC neurons – SCZ microglia + SCZ neurons – SCZ microglia + HC neurons – HC microglia + SCZ neurons	– Microglia derived from blood monocytes – iPSC-derived neurons	– Phagocytosis assay – ICC – Complement depositon assay	↑ co-localizing C4 and PSD95 inclusions	↑ expression of C4	↑ engulfment of synaptic structures ↑ pre- and post-synaptic uptake ↑ expression of C4	Minocycline treatment of SCZ microglia before co-culturing	Sellgren et al., 2019
**Co-culture microglia + NGN2 neurons:** – HC microglia + HC neurons – SCZ microglia + SCZ neurons – SCZ microglia + HC neurons – HC microglia + SCZ neurons	– iPSC-derived microglia – iPSC-derived neurons	– ICC	↑ IBA1 expression ↑ NLRP2, NLRP3, TLR4 ↑ C4A ↑ NFkB signaling ↑ inflammasome activation	↓ presynaptic density ↓ synapse density	↑ presynaptic uptake in microglia ↑ IBA1 expression of microglia ↓ presynaptic density in neurons	Minocycline treatment of SCZ microglia before co-culturing	Breitmeyer et al., 2023
**Co-culture microglia + cortical interneurons:** – unstimulated microglia + HC neurons – stimulated microglia + HC neurons – unstimulated microglia + SCZ neurons – stimulated microglia + SCZ neurons	– BV2 murine microglia – iPSC-derived neurons	– RNA sequencing – Pathway analysis	Not reported	Not reported	↑ inflammatory genes in neurons ↑ involvement of metabolic pathway in neurons ↓ mitcohondiral function of neurons	Activation of BV2 microglia via 1 mg/ml LPS + 1 mg/ml polyI-C before co-culturing	Park et al., 2020

### 4.3 Minocycline application as anti-inflammatory treatment

Findings in patient studies, rodent studies and iPSC studies revealed an increased inflammation status linked to SCZ, hence bringing into question an anti-inflammatory treatment. In both of the two described iPSC-derived co-culture models, pre-treatment of SCZ microglia cells with minocycline before co-culturing with neurons resulted in decreased microglial engulfment of synaptic structures and recovered the presynaptic density, as well as showing a decreasing effect in the activation of SCZ microglia (Sellgren et al., 2019; Breitmeyer et al., 2023). Previous studies in mice already demonstrated the anti-inflammatory effect of minocycline which seems to be beneficial in several neurodevelopmental and neurodegenerative diseases (Monte et al., 2013). The rescuing effect observed in MIA offspring treated with the antibiotic described in the section above, already suggested a link between activation of microglia induced by inflammation and resulting neuronal abnormalities (Xia et al., 2020). A neuroprotective effect of minocycline has also been observed against excitotoxicity, an effect that has been implicated in the disease pathophysiology of neuropsychiatric diseases (Dean et al., 2012). Although anti-inflammatory treatment in iPSC and rodent studies shows promising effects in rescueing the neuronal phenotypes, there is no evidence for beneficial effects of minocycline in patient studies. However, anti-inflammatory treatment approaches in patient studies need more detailed clinical stratification, especially by focusing on disease onset and inflammatory status of patients. As described in the section 2.1 and 2.3, a major problem is the huge variety between the inflammation status of SCZ patients, since not all of them show up elevated inflammatory markers and respond to anti-inflammatory treatments. Hence, it remains difficult to find the adequate treatment and pinpoints the need for a more personalized medicine approach, as it is possible with patient-derived iPSC.

## 5 Critical evaluation and future perspectives

**FIGURE 1:**
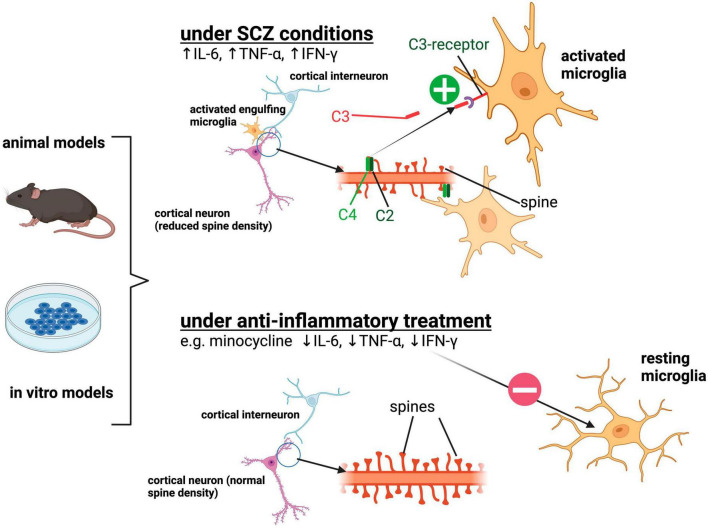
Summarization of the previously described findings implicating aberrant synaptic pruning by activated microglia in SCZ. Under SCZ conditions, microglia depict an activated state morphologically and functionally by the release of pro-inflammatory cytokines such as IL-6, TNF-α, IFN-γ. Activated microglia show increased engulfment of synapses and spines of cortical pyramidal neurons resulting in reduced synaptic density. Increased expression of the complement protein C4A in SCZ neuronal structures is assumed to be responsible for the excessive removal of synapses via the complement system. Anti-inflammatory treatment such as minocycline suppresses the activated microglial phenotype and can lead to reduced uptake of synaptic structures. Created with BioRender.com.

Research conducted in patients and *in vivo* and *in vitro* models has provided evidence of neuroinflammation as one major contributing factor in the pathogenesis of SCZ. Through observations in these model systems, the hypothesis of excess synaptic pruning by activated microglia in a complement-dependent manner has emerged. The major findings supporting the hypothesis are summarized in Figure 1.

### 5.1 Studies of microglia activation in patients

Several methodological limitations related to the study of microglia activation in patients should be pinpointed. When evaluating data from post-mortem studies, one has to bear in mind that cell density may vary between different brain regions (Gober et al., 2022) and drawing conclusions in relation to clinical pathologies can be confounding. Secondly, it is essential to consider the fact that microglia exhibit a flexible nature that can lead to morphological and functional alterations (Caetano et al., 2017). Furthermore, other macrophage types are also stained by Iba-1, which may lead to an overestimation of measurements of microglia density. While additionally most patients have experienced a lifetime of antipsychotic treatment by medication, mostly baring strong cellular effects by itself. Finally, primary causes of death and their effects on microglia by itself cannot be separated from disease specific changes.

One major aspect lies within the analyzed cohorts, which should be designed with a more detailed clinical description of the disease stages and symptoms. Further variability may come from differences in sex, circumstances during disease onset or duration, the effects of medication on the imaging and the existence of different binding-polymorphisms (Turkheimer et al., 2015; De Picker et al., 2017). For the analysis of TSPO levels, variable radiotracers (first and second generation) with different binding affinities have been used, which besides to the application of different methods to calculate TSPO binding, leads to more inconsistency among the published studies (Doorduin et al., 2009; Holmes et al., 2016; Ottoy et al., 2018). As an example, a systematic review on TSPO binding was able to show a significantly elevated binding signal between SCZ patients and controls in 12 studies, but no differences when looking at the volume of distribution (V_*T*_) (Marques et al., 2019). De Picker et al., who performed one of most profound transdiagnostic reviews to date summarizing PET imaging, did not find any significant differences between SCZ cases and controls regarding TSPO PET signal. When analyzing a subgroup of 21 SCZ studies using V_*T*_-based quantification and/or second generation radioligands, there was even a decrease of TSPO signal observed in patients (De Picker et al., 2023). In addition, TSPO is not solely expressed in microglial cells, but also in astroglia und endothelial cells (Notter et al., 2018). Furthermore, only a limited number of studies examined the same parameters in the cohort group of HC, while high-quality longitudinal PET and CSF investigations are still lacking. With a more detailed clinical assessment, bigger sample sizes and stratification some of the above-mentioned issues could be addressed. On the other hand, the employment of highly selective criteria for classification goes in hand with incorporation of a greater number of individuals, presenting a notable limitation in clinical practice.

### 5.2 Sex and age-related differences in microglia-neuron interactions

Besides the need to address the above-mentioned confounding factors, like disease onset or duration and sex in respect to their tracer binding capacity, there also exists an association between age- and sex-dependent susceptibility related to cerebral immune activation, sex hormones, activation of glia cells or cytokine production (Klein and Flanagan, 2016). Transferring this to SCZ appears to be complex. Only looking at the age of onset, recent studies show no differences in sex (Gogtay et al., 2011; Solmi et al., 2022), against former findings describing a delayed age of onset of 3, 2–4 and 1 year for females (Fernando et al., 2020). Nevertheless, addressing sex-specific differences in SCZ, Yu et al. were able to identify a set of microglial genes, specifically deregulated in the female group, using RNA post-mortem data and a MIA model (Yu et al., 2023). More direct findings addressing sex-specific neuron-microglia interactions were mainly performed in rodents using MIA. Hypothesizing sex-specific alterations in microglial pruning activity, a study on adult mice was able to show a sexual dimorphism in synaptic pruning. A female-specific increase in CD68 levels in Iba1-positive microglia and reduced density of excitatory synapses was reported (Hui et al., 2020).

Regarding the effect of age as an influencing factor of microglial activation and neuron-microglia interaction, there is still very limited evidence to date. In one well-conducted longitudinal study, TSPO PET imaging was performed in male psychosis patients (De Picker et al., 2019). Quantifying TSPO uptake based on V_*T*_, the authors report a general increase of relative V_*T*_ with age and an age-dependent difference in V_*T*_ between controls and patients during psychosis. In a second study, Ding et al. investigated age-dependent changes of microglial activation in adolescent and adult MIA-offspring (Ding et al., 2019). They describe a more pronounced upregulation of proinflammatory cytokines in adolescence, preceding the onset of core PPI deficits, suggesting age-dependent changes in inflammation status.

Overall, there is evidence for a role of sex and age-dependent influences on microglial activation in SCZ. However, there is an urgent need to address these aspects in more detail to enable the development of therapies more specifically tailored to e.g., specific disease stages and groups of patients.

### 5.3 Studies of synaptic pruning in model systems

There are still important aspects that need to be addressed regarding microglia-neuron interactions in SCZ. An important point which remains to be explored is the functional consequence of excess synaptic pruning. Multiple lines of evidence implicate E-I imbalance in the cortex of SCZ patients (Gao and Penzes, 2015), but no direct link to cellular phenotypes has been established yet. With respect to E-I imbalance it would be useful to identify whether there is a ‘preferred’ type of synapse undergoing elimination by microglia. So far, most of the studies focused on the interaction between excitatory neurons and microglia (Sellgren et al., 2019; Breitmeyer et al., 2023) whereas it remains elusive whether SCZ microglia do also excessively prune inhibitory synapses. Finally, mechanisms that lead to excessive elimination of synapses are not clearly identified. Patient studies, as well as animal and iPSC studies showed a link to complement protein C4 involved in synaptic pruning, but differences in phagocytosis could not fully be attributed to genetic variations in *C4* (Sellgren et al., 2019). Other proteins that are part of the complement system, such as C1 and C3, have been linked to SCZ, but not studied in detail since the results so far were relatively inconsistent (Mayilyan et al., 2008).

Considering the findings of the above-mentioned PET studies, mainly reporting either no change or a reduction of TSPO binding in SCZ, the question arises how a potential reduction of microglial inflammation would relate to increased synaptic pruning. Corsi-Zuelli and Deakin provided an interesting hypothesis on this matter in a recent review (Corsi-Zuelli and Deakin, 2021). They summarize evidence for elevated astroglial activation and TGF-β signaling in SCZ, which could increasingly convert microglia to a non-inflammatory pruning phenotype.

### 5.4 Microglia-neuron interactions in other neuropsychiatric disorders

Looking beyond SCZ, aberrant neuron-microglia interaction is mainly described for autism spectrum disorder (ASD), while studies of affective disorders as major depression disorder (MDD) or bipolar disorder (BD) report an activation of microglia. In contrast to SCZ, the main hypothesis in ASD postulates the inappropriate removal of less active synapses thus leading to an excess number of synapses, especially excitatory ones (Koyama and Ikegaya, 2015). So far, the most comprehensive work on ASD was done in patients and animal models. Post-mortem tissue analysis of ASD patients revealed increased dendritic spine density in layer V pyramidal neurons in the temporal lobe, as well as the activation and increased density of microglial cells in the prefrontal cortex (Koyama and Ikegaya, 2015; Heider et al., 2021). Increased expression of C1q in the serum of children with ASD, as well as deregulated expression of C4 observed in ASD patients indicate the involvement of the complement system and support the hypothesis of impaired synaptic pruning (Warren et al., 1994; Corbett et al., 2007). While most of the ASD studies hypothesize the contribution of microglia to aberrant synaptic pruning, hence leading to aberrant microglia-neuron interaction, iPSC-based model systems using both microglia and neurons have not been established yet. In MDD, brain imaging studies, as well as animal models show a link between an activation of microglia and depressive symptoms (Wang et al., 2022), while a random clinical trial with patients suffering of a treatment-resistant depression showed no significant changes in MADRS (Montgomery-Asberg Depression Rating Scale) using minocycline as an add-on treatment (Hellmann-Regen et al., 2022). Cerebral inflammation can also be linked to BD, which was done by Haarmann et al. using PET-imaging (Haarman et al., 2014), while on the other hand cellular analysis of post-mortem tissues were not able to show microglial immune activation, as no differences in microglia density nor upregulation of inflammatory markers were observed (Sneeboer et al., 2019).

### 5.5 Limitations and future perspectives of *in vitro* model systems

Animal studies have almost exclusively been conducted in rodents. Although the rodent and human genome present a 95% similarity, one of the major limitations is the lacking complexity of the rodent brain, especially in the cortex (Boroviak et al., 2018). For studies of synaptic pruning, it must be taken into consideration that the rodent complement system is lacking the A and B isoforms of the *C4* gene that depicts one of the major genetic risk variants in SCZ patients (Yilmaz et al., 2021). One of the most important aspects to consider when modeling a disease with iPSC derived from patients is the high variability between patients, therefore large sample sizes are needed to study disease specifics effects. Moreover, iPSC-derived cell types are relatively immature, as they depict embryonic- or fetal-like characteristic (Doss and Sachinidis, 2019).

A future approach to address these open questions is the use of 3D brain organoids that have an improved maturity and complexity compared to traditional 2D iPSC models. There have been several studies on SCZ patient-derived cerebral organoids to recapitulate brain development in a more complex 3D macroenvironment, hence allowing the study of cell type-specific differences in developmental trajectory between CTR and SCZ. While organoids can contain cell types of the ectodermal lineage; microglia usually do not spontaneously arise in these structures since they are of mesodermal origin (Gomez Perdiguero et al., 2015). Therefore, exogeneous microglia must be added externally to the organoid and need to invade and integrate into the existing neuronal circuits (Zhang et al., 2023). However, few studies also showed the generation of brain organoids containing innately developed microglia by using unguided differentiation protocols. This spontaneous formation yielded in highly variable proportion and distribution of microglia (Zhang et al., 2023).

Overall, 3D iPSC-derived organoid models present a promising approach for a more physiological study of inflammation and neuron-microglia interactions in SCZ.

## Author contributions

S-MH: Conceptualization, Visualization, Writing−original draft, Writing−review and editing. JH: Conceptualization, Visualization, Writing−original draft, Writing−review and editing. RW: Conceptualization, Visualization, Writing−original draft, Writing−review and editing. AF: Conceptualization, Validation, Writing−review and editing. HV: Conceptualization, Funding acquisition, Validation, Writing−review and editing.
